# A potassium channel agonist protects hearing function and promotes outer hair cell survival in a mouse model for age-related hearing loss

**DOI:** 10.1038/s41419-022-04915-5

**Published:** 2022-07-11

**Authors:** Barbara Peixoto Pinheiro, Marcus Müller, Michael Bös, Jamil Guezguez, Michael Burnet, Mara Tornincasa, Riccardo Rizzetto, Jean-Francois Rolland, Chiara Liberati, Stefan Lohmer, Youssef Adel, Hubert Löwenheim

**Affiliations:** 1 Translational Hearing Research, Tübingen Hearing Research Center, Department of Otolaryngology, Head & Neck Surgery, University of Tübingen, 72076 Tübingen, Germany; 2 Acousia Therapeutics, 72070 Tübingen, Germany; 3 Synovo, 72076 Tübingen, Germany; 4 Axxam, Bresso, 20091 Milan, Italy

**Keywords:** Drug delivery, Inner ear, Ageing, Cochlea, Hair cell

## Abstract

Age-related hearing loss (ARHL) is the most common sensory impairment mainly caused by degeneration of sensory hair cells in the cochlea with no causal medical treatment available. Auditory function and sensory hair cell survival critically depend on the Kv7.4 (KCNQ4) channel, a voltage-gated potassium channel expressed in outer hair cells (OHCs), with its impaired function or reduced activity previously associated with ARHL. Here, we investigated the effect of a potent small-molecule Kv7.4 agonist on ARHL in the senescence-accelerated mouse prone 8 (SAMP8) model. For the first time in vivo, we show that Kv7.4 activation can significantly reduce age-related threshold shifts of auditory brainstem responses as well as OHC loss in the SAMP8 model. Pharmacological activation of Kv7.4 thus holds great potential as a therapeutic approach for ARHL as well as other hearing impairments related to Kv7.4 function.

## Introduction

According to recent data from the WHO, hearing loss affects 20.3% of the world’s population [1–3]. Age-related hearing loss (ARHL), or presbycusis, has emerged as the leading cause of years lived with disability in people over 70 years of age worldwide, compared to all other disease categories [1, 3]. Although this condition is not considered life-threatening, it can significantly degrade the quality of life and is associated with multiple comorbidities, including social isolation, depression, and cognitive decline [3–5]. Hearing loss has also been suggested as a modifiable risk factor for dementia [6, 7]. Taken together, it is evident that the functional, social, and mental impact, the extraordinary prevalence and burden of disease creates an immense medical need for a causal treatment of ARHL.

In humans, ARHL typically presents as a symmetrical decline in hearing ability over age that is more pronounced in the high frequencies [8, 9]. A recent analysis of human temporal bones showed that the degree of ARHL can be best predicted by the loss of outer hair cells (OHCs) and inner hair cells (IHCs), strongly suggesting sensory presbycusis as the predominant type of ARHL [10]. OHCs provide fast electromechanical amplification of sound and require fast repolarization of the receptor potential in order to respond to the large dynamic range and speed of sound [11]. The modulation of potassium ion (K^+^) channels and potassium circulation in the cochlea maintains this demanding signal transduction process [12, 13]. Specifically, the potassium voltage-gated channel subfamily q member 4 (K_V_7.4 or KCNQ4), which is expressed at the basal pole of OHCs, is involved in K^+^ efflux and generation of the predominant K^+^ conductance current of OHCs, I_K,n_ [14–16].

Impaired surface expression or reduced activity of the K_V_7.4 channel leads to functional impairment and has been associated with age-related [17–21], noise-induced [20, 22–25], ototoxic hearing loss [26], and genetic hearing loss in human hereditary deafness DFNA2 [27–29]. The central role of the K_V_7.4 channel for OHC function and survival has been demonstrated by genetic ablation in *Kcnq4*^−/−^ mice and loss-of-function mutations leading to progressive hearing loss and slow degeneration of OHCs [30, 31]. The loss of the K_V_7.4 in OHCs can result in chronic depolarization, which can consequently lead to their degeneration due to chronic cellular stress [32]. Notably, pharmacological inhibition of K_V_7.4 by linopirdine in an adult guinea pig model has been shown to cause acute hearing loss through compromised function and severe OHC degeneration in the basal turn, which corresponds to the high-frequency range of the cochlea [33]. These various findings lead to the hypothesis that pharmacological activation of K_V_7.4 may preserve hearing function and prevent OHC loss in ARHL, and possibly other forms of hearing loss related to compromised K_V_7.4 function.

For over a decade, K_V_7.4 has been suggested as a drug target for small-molecule activators [20, 26, 34–36]. As a proof of principle, Leitner et al. [36] showed in vitro that synthetic channel openers, such as retigabine (RTG) and zinc pyrithione (ZnP), could potentiate and stabilize the K_V_7.4-mediated I_K,n_ conductance in OHCs with DFNA2-causing mutations in the K_V_7.4 channel. Moreover, they observed an enhancement of the native K_V_7.4-mediated I_K,n_ conductance in OHCs [36]. However, the applicability of small-molecule K_V_7.4 activation as a treatment approach has thus far not been demonstrated in an in vivo model.

In the present study, we investigated the effect of a novel, potent small-molecule K_V_7.4 agonist ACOU085 [37] on ARHL in the senescence-accelerated mouse prone strain 8 (SAMP8) mouse model [38]. The SAMP8 mouse model shows an early progressive, age-related increase in auditory brainstem response (ABR) thresholds as well as loss of OHCs, predominantly in the high-frequency range [18]. The cochlear exposure of ACOU085 from a formulation administered via transtympanic injection was determined in a pharmacokinetic study by analyzing cochlear tissue and perilymph. In an electrophysiology study, the effect of repeated ACOU085 administrations on ARHL was investigated in a within-subject design, with mice receiving unilateral administrations of ACOU085 and contralateral vehicle as control. The magnitude of hearing loss was determined by measuring ABR threshold shifts and showed reduced age-related functional decline in treated ears when compared with vehicle controls. After termination, morphological analysis of cochlear whole-mounts confirmed a concomitant reduction in OHC loss in the high-frequency range of the cochlea. Thus, pharmacological activation of K_V_7.4 appears as an attainable therapeutic approach for ARHL and potentially other hearing impairments related to compromised K_V_7.4 function.

## Methods

### Animals

Female SAMP8/TaHsd mice were acquired from Envigo (Horst, Netherlands) at an age of 30 days and were housed in groups of up to five animals in a standard macrolon polycarbonate cage under a 12-h light-dark cycle with ad libitum access to food and water. Animal care, treatments, and procedures were performed according to the German (TierSchG) and European Union (directive 2010/63/EU) guidelines for the protection of animals used for experimental purposes, following revision and approval by the veterinary care unit of the University of Tübingen and the regional animal care and ethics committee (Regierungspräsidium Tübingen, approval no. HN3/17).

### Drug preparation and administration

ACOU085 (Acousia Therapeutics, Tübingen, Germany) is a novel small-molecule agonist of the K_V_7.4 (KCNQ4) voltage-gated potassium channel with higher potency than ML-213 in the nanomolar range [37] (see Fig. S1). The compound was provided in an injectable formulation which contained proprietary lipid-based gel formulation. In this study, ACOU085 was administered at 0.6% w/v or 6.0% w/v concentrations, with the formulation alone serving as a vehicle control. The formulations were stored at 4 °C and warmed in a water bath to 40 °C before administration.

Transtympanic injection was accomplished by placing the mice in a lateral decubitus position on a custom-made heating pad. The tympanic membrane (TM) was visualized using a surgical microscope (OPMI-1, Zeiss, Oberkochen, Germany). ACOU085 or vehicle formulations were administered into the middle ear until they emerged back through the needle perforation, indicating that the entire middle ear cavity was filled. The applicability of the formulation varied with ACOU085 concentrations; therefore, the 0.6% w/v concentration or the vehicle alone were administered using a 1-ml syringe (B. Braun SE, Melsungen, Germany) and a 20-µl microloader tip (Eppendorf, Wesseling-Berzdorf, Germany). The 6.0% w/v concentration was administered with a 1-ml syringe and a 22 G needle (Epican Paed caudal, Braun, Melsungen, Hessen). For a given mouse, the injection volume was 5–15 µl per ear depending on individual constraints, e.g., anatomical variations, age, and the number of previous transtympanic injections.

### Pharmacokinetics

#### Experimental design

**Fig. 1:**
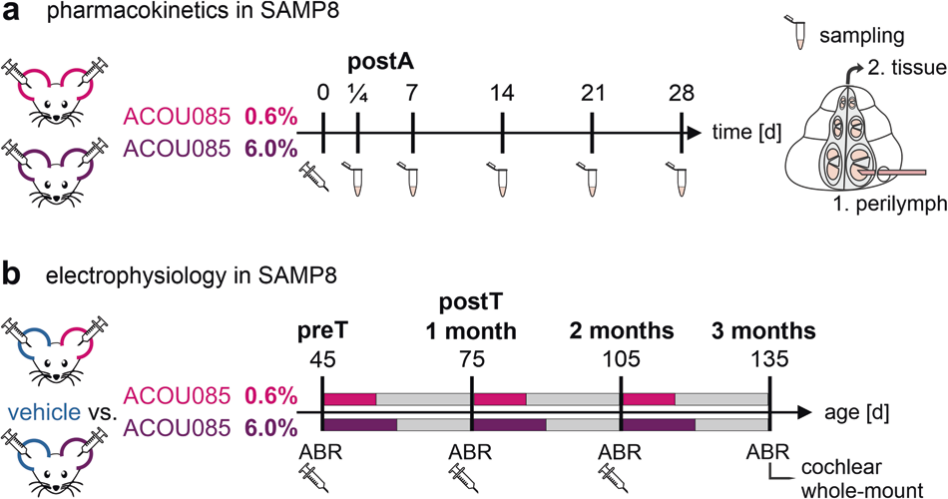
Schematic representation of the pharmacokinetic and electrophysiology study design. **a** Schematic representation of the pharmacokinetic study design. Cochlear perilymph and tissue were sampled after a single administration of 0.6% or 6.0% w/v ACOU085 formulation via transtympanic injection into both ears of SAMP8 mice. Samples were collected for the timepoints 0.25, 7, 14, 21, and 28 days post-administration (postA). Concentrations of ACOU085 in cochlear perilymph and tissue were determined by liquid chromatography and mass spectrometry. **b** Schematic representation of the electrophysiology study design. The effect of repeated unilateral administrations via transtympanic injection of ACOU085 versus contralateral vehicle control was investigated in the SAMP8 mouse model for two dose groups, 0.6% or 6.0% w/v ACOU085, in a within-subject design. Auditory function was assessed at 1-month intervals from the age of 45 days (pre-treatment, preT) to 135 days (3-months post-treatment, postT) by determining auditory brainstem response (ABR) thresholds. After termination, mice were sacrificed and their cochleae were extracted for cochlear whole-mount analysis.

To determine the drug distribution from the middle ear cavity into the cochlea, perilymph and cochlear tissue samples were collected from SAMP8 mice at different timepoints after a single administration via transtympanic injection in each ear of ACOU085 in 0.6% w/v (*n* = 14) or 6.0% w/v (*n* = 24) concentrations. Sampling timepoints were ¼ (6 h), 7, 14, 21, and 28 days (see Fig. 1a). Sample sizes varied between timepoints due to insufficient sampling volumes or contamination. After collection, perilymph and tissue samples were delivered for liquid chromatography with tandem mass spectrometry (LC-MS/MS) analysis.

#### Perilymph and tissue sampling

At the respective timepoint of sample collection, mice were exposed to a gradually increasing amount of CO_2_ until complete cessation of breathing was observed for a minimum of 2 min, followed by decapitation. Temporal bones were extracted from the skull and cochleae were then isolated and dried with a cotton swab prior to sampling to ensure that remnant formulation was not carried into the cochlea during sampling. The perilymph was sampled through the round window by puncturing the membrane with a 20-µl microloader tip (Eppendorf) and collecting a perilymph volume of approximately 2 µl. For tissue sampling, the cochlear apex was perforated with a 27 G needle, then fine forceps were used to widen the opening and collect cochlear tissue. Samples were immediately transferred into PCR tubes (Eppendorf), which were calibrated for weight, after collection. They were stored at 4 °C for a maximum of 2 h before being delivered for LC-MS/MS analysis.

#### Liquid chromatography with tandem mass spectrometry analysis

ACOU085 concentrations in perilymph and cochlear tissue samples were analyzed using LC-MS/MS by Synovo (Tübingen, Germany). Tissue samples were first weighed then approximately five volumes of water were added (generally to a final sample size of 10 mg or 10 µl) followed by a 5-min sonication step as initial preparation. To each perilymph or tissue sample, 6 volumes of acetonitrile (generally 60 µl, Th. Geyer, Renningen, Germany) was added containing the internal injection volume (terbuthylazine). After centrifugation for 5 min at 20,000 RCF, each supernatant was transferred into a glass auto-sampler vial. Dilution of the perilymph or tissue samples was required due to excessive concentration or low sample volume. The dilution factor was considered for data collection and calculations. The processed samples were stored at 4 °C before LC-MS/MS analysis.

All solvents used as mobile phase and for sample preparation were of analytical grade or better. Calibration curves and quality controls were obtained by preparing a stock solution of ACOU085 in DMSO with a final concentration of 10 mM followed by a serial dilution in threefold steps in mouse plasma or artificial perilymph. The calibration curve ranged from 5 to 100,000 nM in final concentration and quality controls had a concentration of 100, 1000, and 10,000 nM. Sample separation was performed using using an Agilent 1260 Binary Pump (Agilent Technologies, Santa Clara, CA, USA), CTC PAL Autosampler (CTC Analytics, Zwingen, Switzerland), and Agilent 1260 thermostatted column compartment (Agilent Technologies). The system was coupled to an API 4500 triple quadrupole mass spectrometer (AB Sciex, Framingham, MA, USA). Data acquisition and processing were performed using Analyst Instrument Control and Data Processing Software (version 1.6.2, AB Sciex).

### Electrophysiology

#### Experimental design

To determine the effect of repeated administrations of a K_V_7.4 agonist ACOU085 on ARHL in the SAMP8 model, two groups of mice received unilateral transtympanic injections of ACOU085 in the right ear in either 0.6% w/v (*n* = 10) or 6.0% w/v (*n* = 10) doses, referred to as the 0.6% or 6.0% groups, respectively. In the contralateral left ears, an equivalent volume of the vehicle formulation was administered as a control. The 0.6% and 6.0% groups were tested independently in consecutive experimental series. At the age of 45 days, preT ABR measurements were conducted, followed by initial treatment via transtympanic injection of ACOU085 or vehicle. Due to asymmetrical hearing loss in preT ABR measurements, two mice of the group treated with 6.0% ACOU085 had to be excluded, yielding a sample size of *n* = 8 for this group. Two subsequent administrations followed in 1-month intervals, i.e., at 75 and 105 days of age. Follow-up ABR measurements were conducted at 1-month, 2-months, and 3-months post-treatment timepoints (Fig. 1b). After the last follow-up ABR measurement at 3-months post-treatment (age of 135 days), cochleae were extracted for immunohistochemical cochlear whole-mount analysis.

#### Auditory brainstem response

ABR was recorded in response to click (100-µs square pulse) or tonebursts at frequencies 2.0–45.2 kHz in two steps per octave, with a duration of 3 ms and 1 ms rise and fall times. Stimuli were presented at 10–100 dB SPL in 3 dB steps with alternating condensation and rarefaction polarities. To generate the stimuli and record the ABR signal, a multi-function I/O-card (National Instruments, Austin, Texas, USA) was used. Acoustic stimuli were delivered in a calibrated open-field system using a dynamic loudspeaker placed lateral to the respective auricle of the mouse. The sound pressure level was calibrated before each block of measurements with a microphone probe (Brüel & Kjær Types 4939 and 2670, Nærum, Denmark) placed near the entrance of the external auditory canal in line with the loudspeaker at an angle of 90°. A differential amplifier recorded the ABR signal between silver wire electrodes inserted subcutaneously at the back (ground), the vertex (positive terminal), and at the mastoid of each ear (negative terminal) with 80 dB gain. Signals were filtered between 100 Hz and 5 kHz using sixth-order Butterworth low- and high-pass filters and then processed by the Audiology Lab software (Otoconsult, Frankfurt am Main, Germany) after analog-to-digital conversion at 50 kHz sampling frequency. ABR measurements were conducted in a soundproof chamber (IAC Acoustics, Niederkrüchten, Germany). An ABR threshold was defined as the sound pressure level at which a stimulus-related response was clearly identified by visual inspection of the ABR signal averaged from 128 stimulus repetitions for each polarity.

All animals were anesthetized during ABR measurements by intraperitoneal injection of 0.05 mg/kg fentanyl (Fentadon, Dechra, Aulendorf, Germany), 0.5 mg/kg medetomidine hydrochloride (Dormilan, alfavet, Neumünster, Germany) and 2.5 mg/kg midazolam (Hameln Pharma, Hameln, Germany). To preserve eye moisture, an ointment (Bepanthen, Bayer AG, Leverkusen, Germany) was administered. Over the course of the measurements, the animals were placed in the prone position, electrocardiography was monitored, and a heating blanket maintained their body temperature at ~37 °C.

#### Cochlear whole-mount analysis

Following ABR measurement at 3 months post-treatment (135 days of age), pre-anesthetized mice from both experimental groups were sacrificed by intracardiac injection of 600 mg/kg pentobarbital sodium (Narcoren, Boehringer Ingelheim, Ingelheim am Rhein, Germany), followed by decapitation. Temporal bones were extracted, dissected on ice, perfused with 4% formaldehyde, and decalcified in 0.2 M EDTA for 27 h at 4 °C. Once decalcification was completed, the cochlear sensory epithelium, i.e., the Organ of Corti (OC), was dissected under a stereo microscope (Zeiss Stemi 200-C). OC extraction was performed by removing the bony labyrinth, detaching the stria vascularis (SV), then separating the OC from the spiral ganglion. The OC was then divided into three segments: apical, middle, or basal. For each cochlea, the three OC segments were finally transferred into one well of a 48-well plate filled with 500 µl of PBS.

For fluorescence and immunofluorescence labeling, whole-mount preparations were first permeabilized with 0.2% Triton X-100 in PBS for 20 min and immersed in a blocking buffer containing 0.2% Triton X-100 in PBS and 1% normal donkey serum for 30 min at room temperature. Visualization was achieved by incubation with anti-myosin VIIa (1:400, rabbit, Proteus Biosciences Inc., Waltham, MA, USA), followed by detection with an Alexa 488-conjugated anti-rabbit secondary antibody (1:400, Invitrogen, Paisley, UK). Each antibody was diluted in PBS supplemented with 0.2% Triton X-100 and 0.5% normal donkey serum. All samples were incubated for 20 min at room temperature in DAPI (1:100, Sigma-Aldrich, St. Louis, MO, USA) and phalloidin 568 (1:400, Invitrogen) for nuclear and F-actin fluorescence staining, respectively, then coverslipped using FluorSave mounting medium (Calbiochem, Merck, Darmstadt, Germany). Imaging of the immunolabelled whole-mount preparations was conducted with 10x magnification using an epifluorescence microscope (Zeiss Axioplan 2 with an ApoTome.2 unit).

### Data analysis

#### Cytocochleograms

Images of OC whole-mount preparations were analyzed using ImageJ (NIH, Bethesda, MD, USA) and cell counting was carried out manually by assigning different counting markers stored with their coordinates using the plug-in “Cellcounter”. The length of each OC whole-mount segment (apical, middle, or basal) was measured along the clearly defined junction connecting the outer pillar cells and the first row of OHCs. Along the longitudinal axis of the OC, this line was traced and concatenated across all three segments to determine the total spiral length. OHCs and IHCs were counted as present if co-staining of three cellular markers were evident, i.e., the nucleus (DAPI), the stereocilia bundle (phalloidin), and the cytoplasm (myosin VIIa). If any of these cellular markers were absent, hair cells were counted as missing. For each counting marker (present or missing OHCs and IHCs), the nearest point on the spiral of the OC was assigned by calculating the minimum Euclidean distance. The total spiral length was normalized to 100% and the number of present or missing OHCs and IHCs, respectively, was calculated as a function of the relative distance from the apex subdivided into 5% bins. This approach was used to determine the extent and pattern of cell loss along the cochlear length mapped as a “cytocochleogram” [18, 39, 40].

#### Statistics

Data are presented as means with standard deviations (SD), with standard error of the mean (SEM), or as box plots. Sample distributions were tested for normality by the Shapiro-Wilk test. Within-subject differences were compared for statistical significance using the two-sided paired-samples *t*-test for two samples, or two-way repeated-measures analysis of variance (ANOVA) for more than two samples to determine the main effect of treatment. A *p*-value of less than 0.05 was considered statistically significant and, if applicable, was adjusted for multiple comparisons using the Bonferroni correction. Statistical analysis was performed using IBM SPSS Statistics 27 (IBM Corporation, Armonk, NY, USA).

## Results

### ACOU085 readily diffuses into the cochlea from the middle ear cavity

**Fig. 2:**
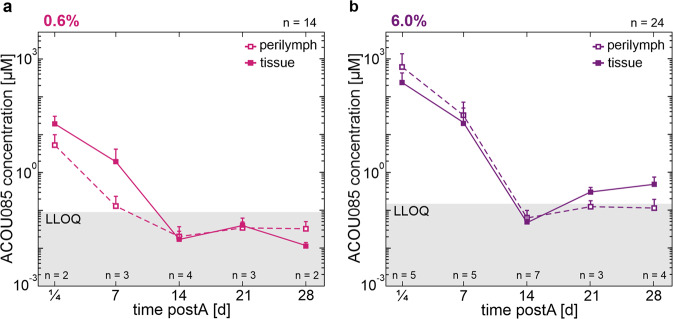
Distribution of ACOU085 in the cochlea of SAMP8 mice after a single administration. Concentrations of ACOU085 in cochlear perilymph and tissue were determined by liquid chromatography with tandem mass spectrometry (LC-MS/MS). Mean and standard deviation of ACOU085 concentrations are shown for perilymph (open squares) and tissue (closed squares) sampling after a single administration of the 0.6% (**a**) or 6.0% (**b**) w/v ACOU085 formulation. The lower limit of quantification (LLOQ) was calculated for each timepoint taking dilution factors into account, with the mean LLOQ over all timepoints shown (gray shading).

To determine the diffusion of the K_V_7.4 activator ACOU085 from the formulation administered into the middle ear cavity into the targeted cochlea, the concentration of ACOU085 was sampled in cochlear perilymph and tissue for the timepoints ¼ (6 h), 7, 14, 21, and 28 days after a single transtympanic injection of 0.6% w/v (*n* = 14) or 6.0% w/v (*n* = 24) formulations in SAMP8 mice (Fig. 1a). For each mouse, ACOU085 concentrations obtained by LC-MS/MS for either perilymph or tissue samples were averaged, respectively, across both ears. The lower limit of quantification (LLOQ) was averaged from individual LLOQs that were calculated for each timepoint taking dilution factors into account, with the mean LLOQ over all timepoints shown in gray shading (Fig. 2a and b). For the 0.6% w/v ACOU085 formulation, the mean concentration measured in perilymph at ¼ days post-administration (postA) was 5.20 ± 4.54 µM, whereas the concentration in cochlear tissue reached more than thrice that level with a mean concentration of 18.86 ± 11.03 µM (Fig. 2a). At 7 days postA, the concentration decreased to 0.13 ± 0.10 µM in perilymph and to 1.89 ± 2.16 µM in tissue. At 14 days and beyond, concentrations in both sample types were found below LLOQ. For the 6.0% w/v formulation, at ¼ days postA a mean ACOU085 concentration of 621.45 ± 754.15 µM was found in the perilymph, and 247.25 ± 181.34 µM in tissue (Fig. 2b). These concentrations are at least an order of magnitude higher than for the 0.6% w/v formulation, which is consistent with a dose-dependent exposure [41]. At 7 days postA, mean ACOU085 concentration in the perilymph remained relatively high at 32.72 ± 39.77 µM, and in the tissue, it was 21.12 ± 29.58 µM. Similar to the 0.6% w/v formulation, ACOU085 concentrations at 14 days and beyond were generally below or at LLOQ for the 6.0% w/v formulation. However, tissue concentrations appeared to increase slightly at 21 days and 28 days postA (Fig. 2b). This may be due to variability between mice given that sampling was terminal and not continuous within subjects. In summary, this pharmacokinetic study demonstrated that ACOU085 readily diffused into the cochlear perilymph and tissue from the middle ear cavity in a dose-dependent fashion. Given an EC_50_ in the nanomolar range [37] (Fig. S1), therapeutically relevant concentrations of ACOU085 are estimated to be available in the cochlea for 7–14 days after a single administration, with higher concentrations and a presumably longer time window for the 6.0% w/v dose.

### K_V_7.4 agonist reduces age-related ABR threshold shifts in SAMP8 mice

**Fig. 3:**
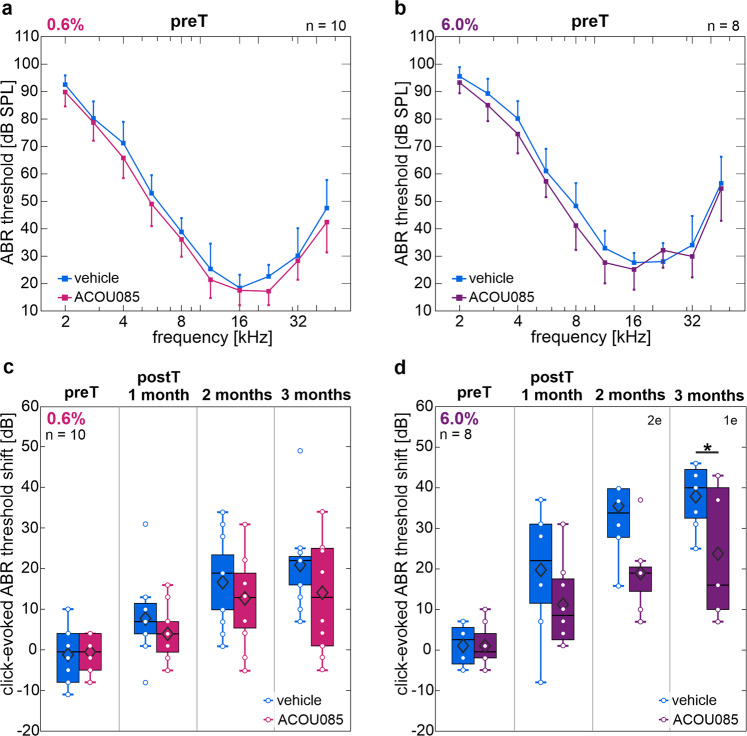
ABR thresholds of SAMP8 mice before treatment and click-evoked ABR threshold shifts of SAMP8 mice treated with ACOU085 and vehicle control. **a**, **b** Mean and standard deviation of ABR thresholds are shown for SAMP8 mice at the age of 45 days before treatment with ACOU085 at 0.6% (**a**) or 6.0% (**b**) w/v dose or contralateral vehicle control. **c**, **d** Means (diamonds), box plots, and individual (circles) click-evoked auditory brainstem response (ABR) threshold shifts are shown for SAMP8 mice treated unilaterally with either 0.6% (**c**) or 6.0% (**d**) w/v ACOU085 and contralateral vehicle control measured at different timepoints: pre-treatment (preT), and 1-, 2-, 3-months post-treatment (postT). Threshold shifts were calculated as the difference between individual thresholds and the population mean at preT. Significant pairwise comparisons (paired-samples *t-*Test) are indicated by asterisks (*p < 0.05). Note that due to technical issues resulting in data loss, two mice had to be excluded at the 2-months and 1 mouse at the 3-months postT intervals, respectively, in the 6.0% group. This is denoted by “1e” and “2e”, i.e., 1 ear and 2 ears excluded, respectively.

The pharmacodynamic effect of cochlear K_V_7.4 enhancement on ARHL in the SAMP8 model was investigated in two groups of mice that received three consecutive, unilateral transtympanic injections of ACOU085 in 0.6% w/v (*n* = 10) or 6.0% w/v (*n* = 8) doses, hereafter referred to as the 0.6% or 6.0% group, respectively. In contralateral ears, equivalent volumes of the vehicle formulation were administered as a control. For each group, the baseline measurement, i.e., pre-treatment (preT) ABR thresholds, was conducted at the age of 45 days followed by initial administration via transtympanic injection of ACOU085 or vehicle. Two subsequent administrations followed in 1-month intervals, i.e., at 75 and 105 days of age. Based on the pharmacokinetic study, we estimate that at least half the 1-month administration interval had sub-EC_50_ concentrations of ACOU085 in the target tissue for either group, suggesting that effects were obtained without sustained target engagement. Follow-up ABR measurements were conducted at 1-month, 2-months, and 3-months post-treatment (postT) intervals (Fig. 1b). At preT, toneburst- (Fig. 3a, b) and click-evoked (Fig. 3c, d) ABR thresholds showed no statistically significant differences between ears selected for treatment with ACOU085 or vehicle in either group. ABR threshold shifts were calculated as the difference between individual thresholds (for each ear) and the population mean at preT (*n* = 36, two ears per mouse). In general, the 6.0% group showed a more rapid progression of hearing loss compared with the 0.6% group (Fig. 3c, d); when comparing click-evoked threshold shifts of vehicle-treated ears between these groups at 3-months postT, the mean threshold shift for the 0.6% group was 21.0 ± 11.6 dB in contrast to a 37.9 ± 8.1 dB threshold shift for the 6.0% group. These significantly different threshold shifts (*p* = 0.0048) between vehicle-treated ears may be traced back to the variability of age-related threshold loss in SAMP8 mice [18]. However, this does not affect within-subject comparisons in either dose group. Within-subject comparisons of click-evoked ABR threshold shifts in the 0.6% group showed no significant effect of treatment for any postT interval, but a trend of decreased threshold shifts for ACOU085- compared with vehicle-treated ears can be observed (Fig. 3c). In the 6.0% group, a similar trend was observed and significant within-subject differences between ACOU085- and vehicle-treated ears were evident at 3-months postT (*p* = 0.029, Fig. 3d). Tone-burst evoked ABR also showed similar trends for both dose groups (see Fig. S2); the main effect of treatment (ACOU085 vs. vehicle) at 3-months postT was statistically significant in the 0.6% group, *F*(1,9) = 11.76, *p* = 0.008, and was just above significance level in the 6.0% group, *F*(1,6) = 5.596, *p* = 0.056. Altogether, the reduced ABR threshold shifts demonstrate that repeated treatments with the K_V_7.4 agonist protected hearing function from the age-related decline in SAMP8 mice.

### K_V_7.4 agonist reduces age-related OHC loss in SAMP8 mice

**Fig. 4:**
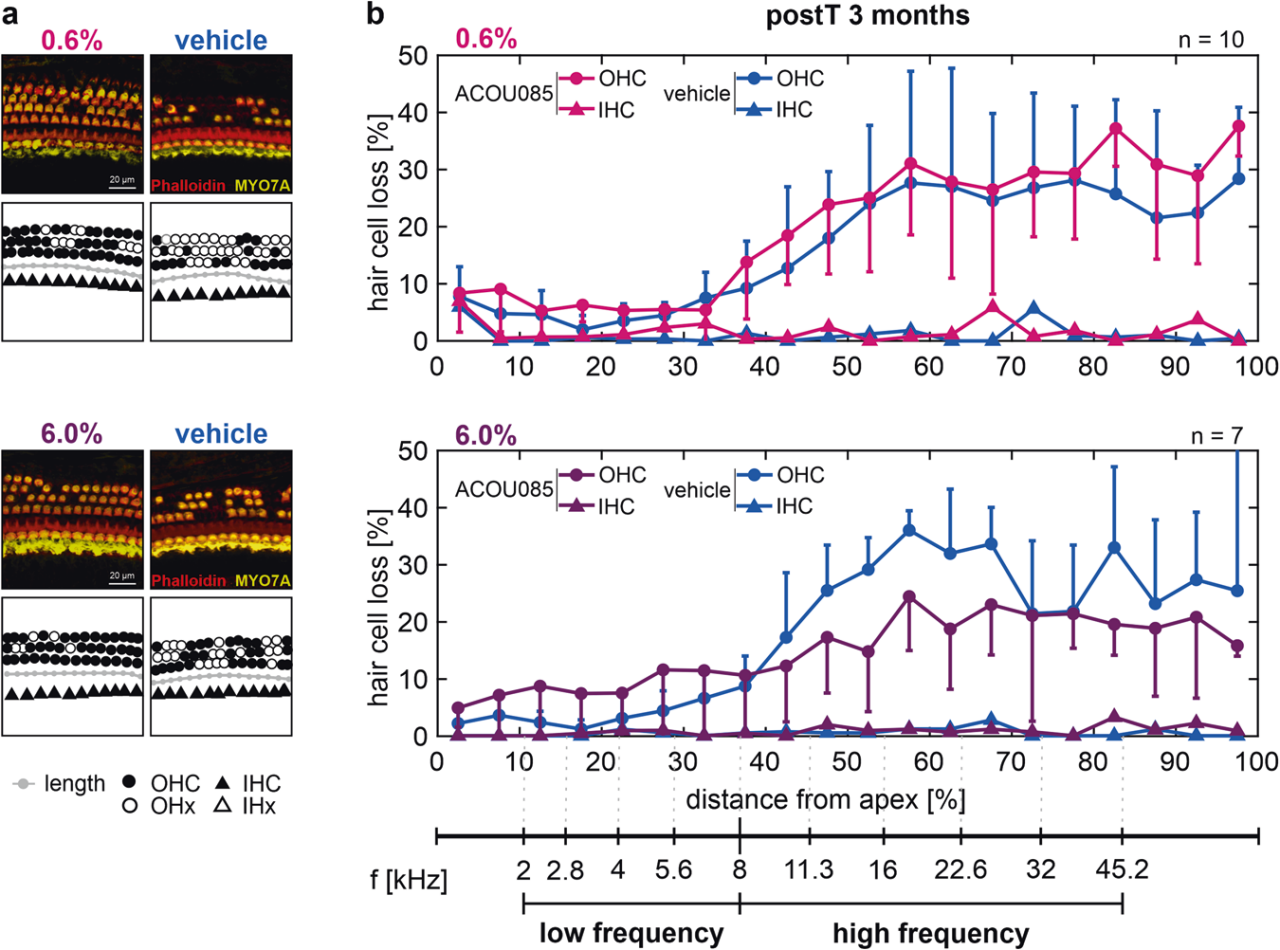
Quantification of outer and inner hair cell loss in SAMP8 mice treated with ACOU085 and vehicle control. The effect of repeated unilateral administrations via transtympanic injection of ACOU085 versus contralateral vehicle control was investigated in the SAMP8 mouse model in a within-subject design. After the last follow-up measurement at 3-months post-treatment (postT, age of 135 days, see Fig. 1b), cochleae were extracted for immunohistochemical analysis to determine the degree of outer and inner hair cell (OHC and IHC, respectively) loss for either 0.6% or 6.0% w/v ACOU085 and corresponding vehicle control. **a** Illustrative examples are shown for cochlear segments (scale bar, 20 µm) stained with myosin VIIa (MYO7A, yellow) for cytoplasm and phalloidin (red) for stereocilia of SAMP8 mice treated with either 0.6% or 6.0% w/v ACOU085 and vehicle control. **b** Cytocochleograms show mean and standard error of the mean OHC and IHC loss in percent, respectively, which are calculated as the ratio of absent hair cells to the sum of present and absent hair cells within 5% bins of distance from apex, for the 0.6% (*n* = 10) or the 6.0% group (*n* = 7, due to 1 exclusion at 3-months postT). A mouse place-frequency map [39, 40, 55] was then used to divide the cytocochleograms into low- (≤8 kHz) and high-frequency (>8 kHz) ranges. For reference, a frequency (*f*) axis is depicted with respect to the relative distance from apex.

**Fig. 5:**
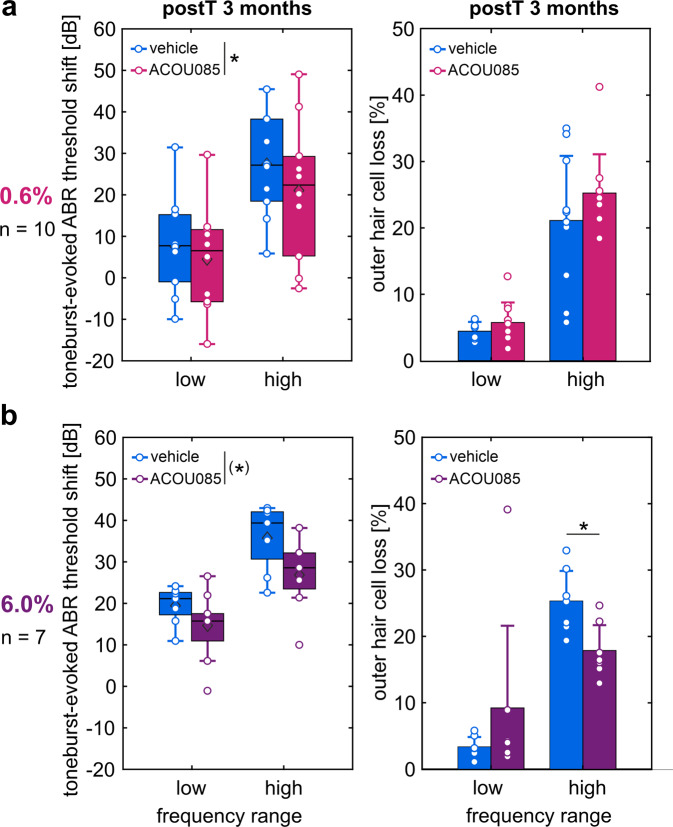
Repeated treatments with K_V_7.4 agonist reduced age-related decline of toneburst-evoked ABR threshold shifts and age-related OHC loss in the high-frequency range. Following the final follow-up measurement at 3-months post-treatment (postT, age of 135 days, see Fig. 1b), cochleae were extracted for immunohistochemical analysis to generate cytocochleograms. A mouse place-frequency map was used to divide the cytocochleograms into low- (≤8 kHz) and high-frequency (>8 kHz) ranges (see Fig. 4b), then outer hair cell (OHC) loss was averaged over each frequency range. Correspondingly, toneburst-evoked auditory brainstem response (ABR) threshold shifts at 3-months postT were averaged over each frequency range, for each mouse and treatment. Means (diamonds), box plots, and individual (circles) toneburst-evoked ABR threshold shifts are compared with mean and standard deviation of OHC loss of SAMP8 mice treated unilaterally with ACOU085 at 0.6% (**a**, *n* = 10) or 6.0% w/v dose (**b**, *n* = 7, due to 1 exclusion at 3-months postT) and contralateral vehicle control. A significant main effect of treatment (ACOU085 vs. vehicle, two-way repeated-measures ANOVA) and significant pairwise comparisons (two-tailed paired-samples *t*-test with Bonferroni correction for multiple comparisons) are indicated by asterisks (*p < 0.05). The main effect of treatment just above the significance level (*p* = 0.056) is indicated by an asterisk within parentheses.

To determine the effect of treatment on the degree of hair cell survival, cochleae were extracted for immunohistochemical analysis after the final follow-up ABR measurement at 3-months postT. Cytocochleograms were generated from OC whole-mount preparations stained for the nucleus (DAPI), the stereocilia bundle (phalloidin), and the cytoplasm (myosin VIIa). Illustrative examples of OC whole-mount analysis are shown in Fig. 4a. Both present and absent OHCs and IHCs were respectively counted and divided into 5% bins along the normalized spiral length of the OC as previously described [18]. Cytocochleograms visualize the percentage of hair cell loss in each bin based on the ratio of absent hair cells to the sum of present and absent hair cells (Fig. 4b). In both the 0.6% and 6.0% groups, IHC loss remained negligible below 7% for either treatment. This finding is consistent with previous histological data on age-related IHC loss in SAMP8 mice [18, 42]. As is also expected from previous data, mean OHC loss of up to 36% was observed in the high-frequency range of vehicle-treated ears, starting at a distance of 30–40% from the apex. OHC loss showed no relevant differences between ACOU085- and vehicle-treated ears in the 0.6% group. By contrast, OHC loss in the 6.0% group was generally reduced up to 14% in ACOU085-treated ears. Given that cytocochleogram bins without valid hair cell counting markers for a given cochlea had to be excluded from the analysis (see Methods), statistical comparisons between treatments were not possible for each bin. A mouse place-frequency map was implemented to convert the relative distance from apex to corresponding center frequencies [39], which facilitates a more direct comparison with ABR data. According to the previously shown progressive OHC loss in SAMP8 mice in the middle and basal regions (>40% distance from apex) in contrast to the apical region, the place-frequency map was used to divide cytocochleograms into low- (≤8 kHz) and high-frequency (>8 kHz) ranges. OHC loss was then averaged over each frequency range. Correspondingly, toneburst-evoked ABR threshold shifts at 3-months postT were averaged over the same frequency ranges (low or high), for each treatment and group to allow frequency range-specific comparisons with OHC loss. The 0.6% group showed a significant main effect of treatment on ABR threshold shifts with *F*(1,9) = 11.76, *p* = 0.008 (Fig. 5a), which is consistent with the analysis for each frequency at 3-months postT (c.f. Fig. S2a). However, no significant differences were evident in OHC loss. The main effect of treatment on ABR threshold shifts in the 6.0% group was just above significance level, *F*(1,6) = 5.60, *p* = 0.056, which is also consistent with the analysis for each frequency (c.f. Fig. S2b). While no significant main effect of treatment was found on OHC loss, a significant interaction effect of treatment and frequency range was found, *F*(1,6) = 8.43, *p* = 0.027 (Fig. 5b). Post-hoc testing correspondingly showed a significant reduction of OHC loss in ACOU085- compared with vehicle-treated ears in the high-frequency range (*p* = 0.00063). This frequency range-specific effect of treatment with reduced OHC loss is consistent with the significant reduction in click-evoked ABR threshold shifts at 3-months postT (Fig. 3d). In summary, repeated administration of the K_V_7.4 agonist in the 6.0% group over a period of three months was shown to significantly reduce age-related OHC loss in the high-frequency range. This increased morphological preservation of OHCs correlated with the functional protection of ABR thresholds in this group.

## Discussion

Hearing impairment is the third most common sensory deficit in humans, with ARHL constituting the leading cause in the population older than 70 years [3]. Causal and effective medical treatments for ARHL constitute a significant unmet need in the elderly. Dysfunctional K_V_7.4 has been previously associated with genetic, noise-induced, and age-related hearing loss [19, 22, 24]. Therefore, maintaining K_V_7.4 expression in OHCs was suggested as a promising therapeutic approach for hearing loss. In the present study, we examined a treatment principle for ARHL by K_V_7.4 channel activation, which has a central role for OHC function and survival [30, 31]. A potent small-molecule K_V_7.4 agonist (ACOU085 [37]) was shown to diffuse from a transtympanically injected formulation into the cochlea, and had a protective effect on age-related ABR threshold shifts and OHC loss in the SAMP8 mouse model of ARHL.

We confirmed in a pharmacokinetic study that ACOU085 administered in a sustained release formulation via transtympanic injection readily diffused from the middle ear cavity into the cochlea. After a single administration of ACOU085 in 0.6% or 6.0% w/v dose, drug levels well above the nanomolar range [37] were reached in cochlear perilymph and tissue. This presumably allowed target engagement within an estimated, therapeutically relevant dose-dependent exposure lasting from 7 to 14 days (c.f. Fig. 2a, b). The administration of a drug formulation through the TM into the middle ear allows drugs to reach the cochlea and has a long-standing clinical application [41]. Transtympanic drug delivery primarily relies on diffusion through the round window membrane (RWM) for drug entry into the cochlea. Although this method presents major challenges for drug delivery, such as presumably unequal distribution over the cochlear spiral or the amount of drug elimination via multiple routes, transtympanic injection as a method for cochlear drug delivery allows for rapid and high local target exposure. Another limitation of transtympanic injection involves the perforation of the TM which can cause scarring that leads to conductive hearing loss. Thus, an administration interval of 1 month was adopted in the electrophysiology study to minimize the effect of TM scarring [43, 44]. Consequently, the therapeutic time window estimated within a time frame of 7–14 days was less than or equal to half of the 1-month treatment interval. This pharmacokinetic restriction posed an additional challenge to the investigated effects of K_V_7.4 activation on age-related decline in the SAMP8 model.

Despite this limited therapeutic time window in the electrophysiology study, a significant protective effect was detected for treatments with ACOU085 in the 6.0% w/v dose at the functional as well as morphological level. In this group, ACOU085 significantly reduced click-evoked ABR threshold shifts when compared with vehicle-treated ears (c.f. Fig. 3d). Auditory thresholds are known to be a sensitive measure of OHC function, as they play an essential role in the lower dynamic range of the cochlear amplifier [45]. The OHC conductance current carried by K^+^ drives their electromotility [46], with K_V_7.4 maintaining the OHC receptor potential and K^+^ homeostasis [14, 15, 33, 47]. A preliminary analysis of the ABR input-output functions of the present study showed no additional suprathreshold effects on wave latency or slope, but only differences in threshold. This suggests that OHCs were the main target of ACOU085 treatment in this model, appearing to have maintained their function and increased their survival rate over age. In line with this assertion, cytocochleograms of the 6.0% group showed significantly reduced OHC loss for ACOU085- compared with vehicle-treated ears in the high-frequency range (>8 kHz, c.f. Fig. 5b), which corresponds to a protective benefit of 29.4%. Although we have not measured the drug distribution along the cochlear length, these data suggest a precedence for protective effects in the basal turn.

The protective effect on the age-related functional decline in SAMP8 was, however, only significant for click-evoked ABR at 3-months postT, but just above significance level for toneburst-evoked ABR in the 6.0% group (see Fig. 3d and Fig. S2b). Acoustic click stimuli generally have a broader spectral spread than transient toneburst stimuli [48], thereby evoking a broader neural population. We have previously observed a large variability between SAMP8 mice in the progression of age-related toneburst-evoked ABR threshold shifts [18]. Thus, the relatively narrow spectral spread and the large variability between mice could have impeded the detection of the protective effects on the local level by toneburst-evoked ABR in the 6.0% group. The opposite observation in the 0.6% group, where ABR threshold shifts evoked by tonebursts were significantly reduced but not for click (see Figs. 3c and 5a), could be traced back to a protective effect dominated by the high-frequency range (≥16 kHz). This is suggested by significant differences in pairwise comparisons for toneburst-evoked ABR at 16 and 32 kHz before adjustment of the significance level for multiple comparisons. Since click stimuli have reduced spectral energy in the region beyond 10 kHz, they would be limited in detecting an effect localized at the frequency range beyond 16 kHz. However, while the functional protection observed in the 6.0% group was in concordance with significantly reduced OHC loss, this was not the case in the 0.6% group. The observed large variability between SAMP8 mice in the progression of age-related threshold decline could previously not be explained by OHC loss alone [18]. Therefore, the protective effect observed in the 0.6% group without reduction of OHC loss can arguably be attributed to a protective effect against the functional sensory degeneration primarily linked to oxidative stress in SAMP8 mice [42, 49, 50].

The survival of OHCs is dependent on the functional K^+^ recycling circuit, which facilitates OHC electromotiliy [51]. An essential component for maintaining K^+^ cycling is the voltage-dependent K^+^ channel K_V_7.1 (KCNQ1), which is expressed in the SV and is responsible for K^+^ secretion to the endolymph. A decrease in K_V_7.1 has been previously observed to cause SV atrophy with notable hearing loss [52]. However, Peixoto Pinheiro et al. [18] have found no consistent correlations between K_V_7.1 membrane expression decline and age in SAMP8 mice. By contrast, relevant linear regressions and negative correlations were found between K_V_7.4 membrane expression in OHCs and age, especially in middle and midbasal turns. This is consistent with the protection of OHC function in both dose groups, as well as OHC survival in the higher dose group. Considering the pharmacokinetic restriction reducing drug exposure to half or less than half of the experimental time and the large variability in age-related auditory decline of the SAMP8 mouse model, the observed protective effects appear very encouraging and have considerable potential for further improvement, e.g., by increased dosing, frequency of treatment, or potentially a different formulation allowing prolonged release of the drug.

Small-molecule K_V_7.4 agonists have been in research for over a decade as a strategy to treat hearing impairments [20, 34]. One of the most characterized K_V_7.4 channel activators is RTG, which causes a shift in the hyperpolarizing direction of the channel’s voltage-dependence [53]. Leitner et al. [36] were the first to show in vitro that a combined administration of ZnP and RTG can functionally rescue K_V_7.4-mediated currents from deafness-causing mutations and, furthermore, this drug combination was able to enhance the native K_V_7.4-mediated I_K,n_ current. An in-vivo study involving K_V_7.4 agonists has only been performed in a rat model of tinnitus, whereby treatment with RTG was able to reverse reduced compound action potential amplitudes at low and high frequencies, respectively [54]. However, RTG failed to reverse reduced distortion-product otoacoustic emissions, suggesting that protection was most probably not mediated at the OHC level. Although the application of K_V_7.4 activators as a treatment modality for ARHL appeared logical from previous K_V_7.4 activation studies [20], the variability of ARHL models and the necessary long-term application have complicated a potential in vivo experimental design to investigate their protective effect. We have demonstrated for the first time in vivo that a novel small-molecule K_V_7.4 agonist can functionally and morphologically protect OHCs in a mouse model of ARHL. These findings suggest that pharmacological K_V_7.4 activation holds great potential as a novel therapeutic approach for ARHL by preventing or decelerating age-related decline of auditory function and morphological loss of OHCs, as well as for other hearing impairments related to compromised K_V_7.4 function.

### Reporting summary

Further information on research design is available in the Nature Research Reporting Summary linked to this article.

## Supplementary information

Supplementary Figures

Figure S1

Figure S2

Reporting Summary

## Data Availability

The data supporting the findings of this study are available from the corresponding authors BP and HL upon reasonable request. Restrictions apply to details, analytics, and formulations of ACOU085 as well as the in-vitro pharmacodynamic data (Fig. S1), which are subject to a non-disclosure agreement with Acousia Therapeutics (Tübingen, Germany). These data can, however, be made available from the corresponding authors upon reasonable request and with permission by Acousia Therapeutics.
