# Supplementary material for: A potassium channel agonist protects hearing function and promotes outer hair cell survival in a mouse model for age-related hearing loss
Source: Cell Death Dis. 2022 Jul 11;13(7):595. doi: 10.1038/s41419-022-04915-5 (PMC9273644; doi:10.1038/s41419-022-04915-5)
Supplement: Supplementary file 1 — Supplementary Figures [file 41419_2022_4915_MOESM1_ESM.pdf]

Figure S1

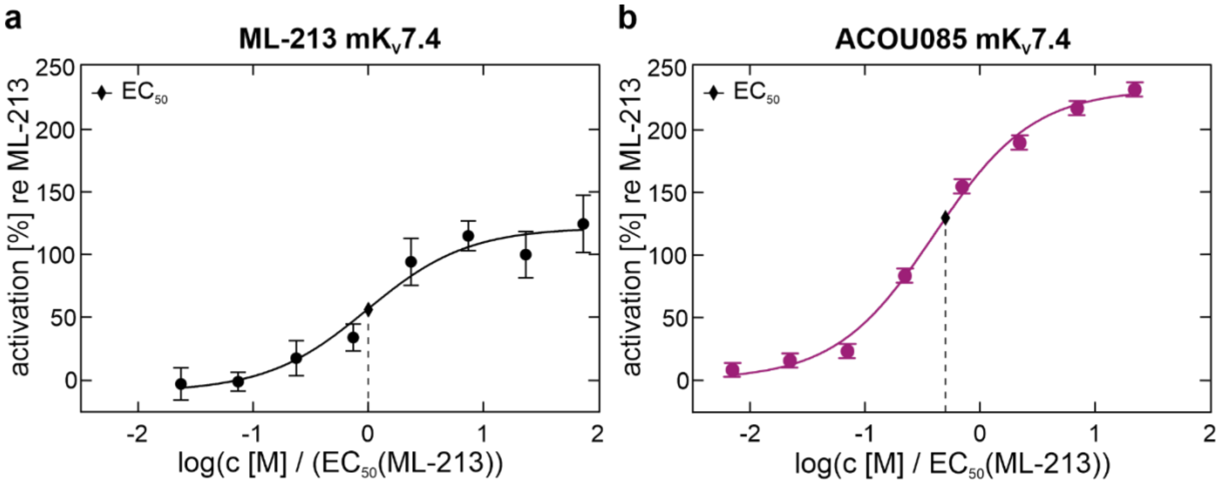

Activity of compounds ML-213 and ACOU085 on CHO-K1 cells expressing mouse $K_V7.4$. The response of Chinese hamster ovary cells (CHO-K1) expressing mouse $K_V7.4$ (mKv7.4 K6.1) was examined for ACOU085 in comparison to the reference channel activator ML-213 (Tocris, Bristol, UK) using FLIPR^{TETRA} Potassium Assay Kits (Molecular Devices, San José, CA, USA) in combination with a fluorescent signal and analyzed by GeneData Screener 14.0 (GeneData, Basel, Switzerland). First, cells were detached from 80-90% confluent flasks and seeded into black-clear 384-MTP in complete medium (25 µl/well) without antibiotics. The medium was removed 24 hours after seeding by manual plate overthrow. Loading of cells was performed in the dark for 1 hour at room temperature with 20 µl/w of 0.5X thallium (Tl^+) dye loading solution consisting of 15 µl Thallos dye reagent and 100 µl Probenecid, which was diluted in 10 ml chloride-free Tyrode's buffer. After incubation with the dye, plates were analyzed by injecting either ACOU085 (n = 4) or the reference activator ML-213 (n = 4) in chloride-free Tyrode's buffer (first injection as 5X, reading 8 minutes) and then injecting chloride-free Tyrode's buffer in presence of Tl^+ (2X solution). This enabled the permeation of Tl^+ through the potassium channel leading to a fluorescence change, which was monitored for 120 seconds. Compound activity was calculated by subtracting the neutral from the stimulator control kinetic, which served as a negative control correction to identify the point of inflection, with which the limits of kinetic analysis were delineated. The effect of the activating compound is expressed as a percentage of activation, with 100% activation normalized to the response value of ML-213 at EC_{100} plus EC_{20}. The neutral control corresponded to EC_{20} of Tl^+ alone. Mean and standard deviation of percentage activation are shown for the response to ML-213 (a) and ACOU085 (b) as a function of the logarithmic concentration with respect to the calculated ML-213 EC_{50} value, which is in the nanomolar range. ACOU085 showed more than 2-fold higher activation and was approximately 2.5-fold more potent than ML-213.

Figure S2

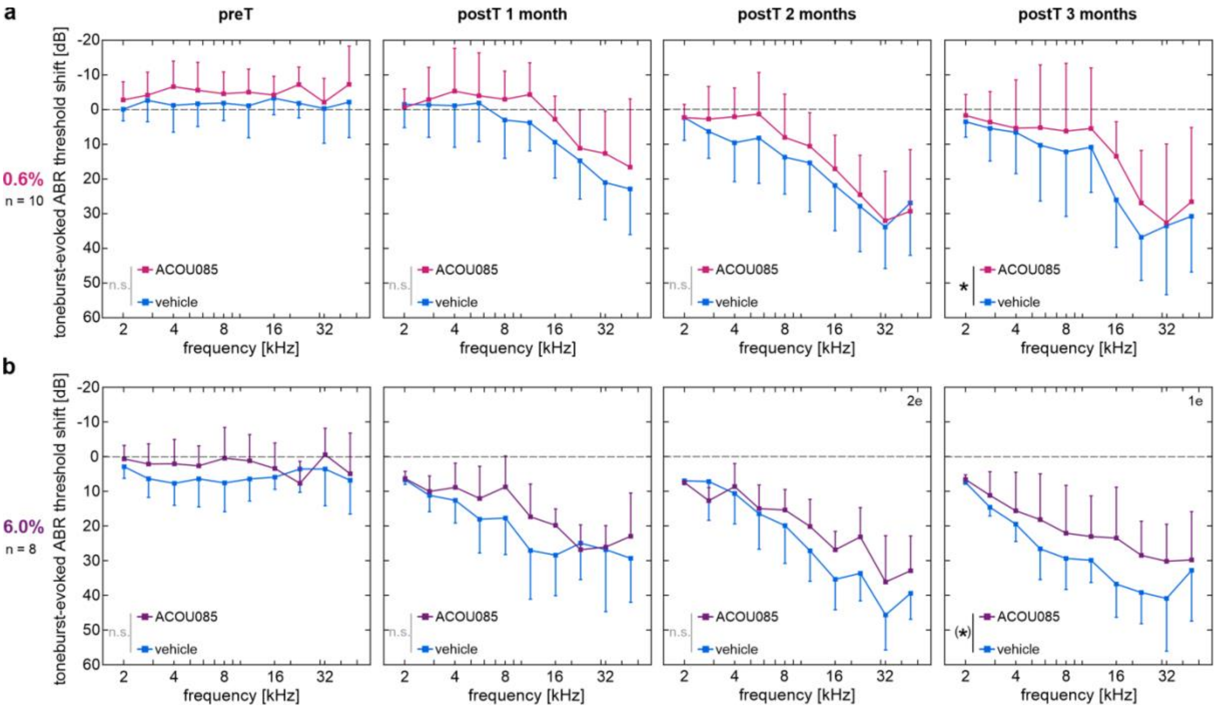

Toneburst-evoked ABR threshold shifts of SAMP8 mice treated with ACOU085 and vehicle control. Mean and standard deviation of toneburst-evoked auditory brainstem response (ABR) threshold shifts are shown for SAMP8 mice treated unilaterally with either 0.6% w/v (a, n = 10) or 6.0% w/v (b, n = 8) ACOU085 ($K_V7.4$ agonist) with contralateral vehicle control, which were measured at timepoints: pre-treatment (preT), 1-, 2-, and 3-months post-treatment (postT). Toneburst-evoked ABR threshold shifts were calculated as the difference between individual thresholds (for each ear) and the population mean at preT (n = 36, two ears per mouse). A significant main effect of treatment (ACOU085 vs. vehicle, two-way repeated-measures ANOVA) is indicated by an asterisk (*, $p < 0.05$). At 3-months postT, the main effect of treatment was statistically significant in the 0.6% group, $F(1,9) = 11.76$, $p = 0.008$, but was just above significance level in the 6.0% group, $F(1,6) = 5.596$, $p = 0.056$ as is indicated by an asterisk in parentheses. Note that due to technical issues resulting in data loss, two mice had to be excluded at the 2-months and 1 mouse at the 3-months postT intervals, respectively, in the 6.0% group. This is denoted by “1e” and “2e”, i.e., 1 ear and 2 ears excluded, respectively. n.s., not significant.
