# Supplementary figures and images for: A potassium channel agonist protects hearing function and promotes outer hair cell survival in a mouse model for age-related hearing loss
Source: Cell Death Dis. 2022 Jul 11;13(7):595. doi: 10.1038/s41419-022-04915-5 (PMC9273644; doi:10.1038/s41419-022-04915-5)

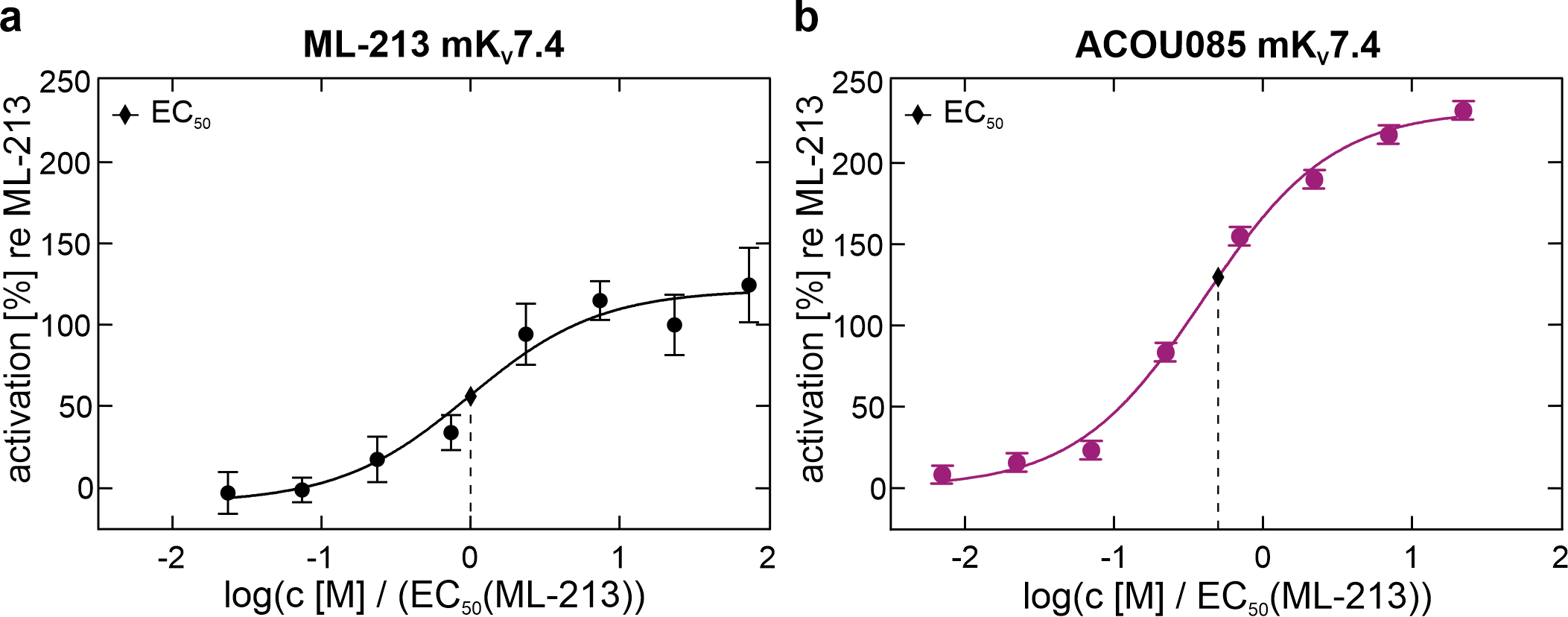

Supplement: Supplementary file 2 — Figure S1 [file 41419_2022_4915_MOESM2_ESM.png]

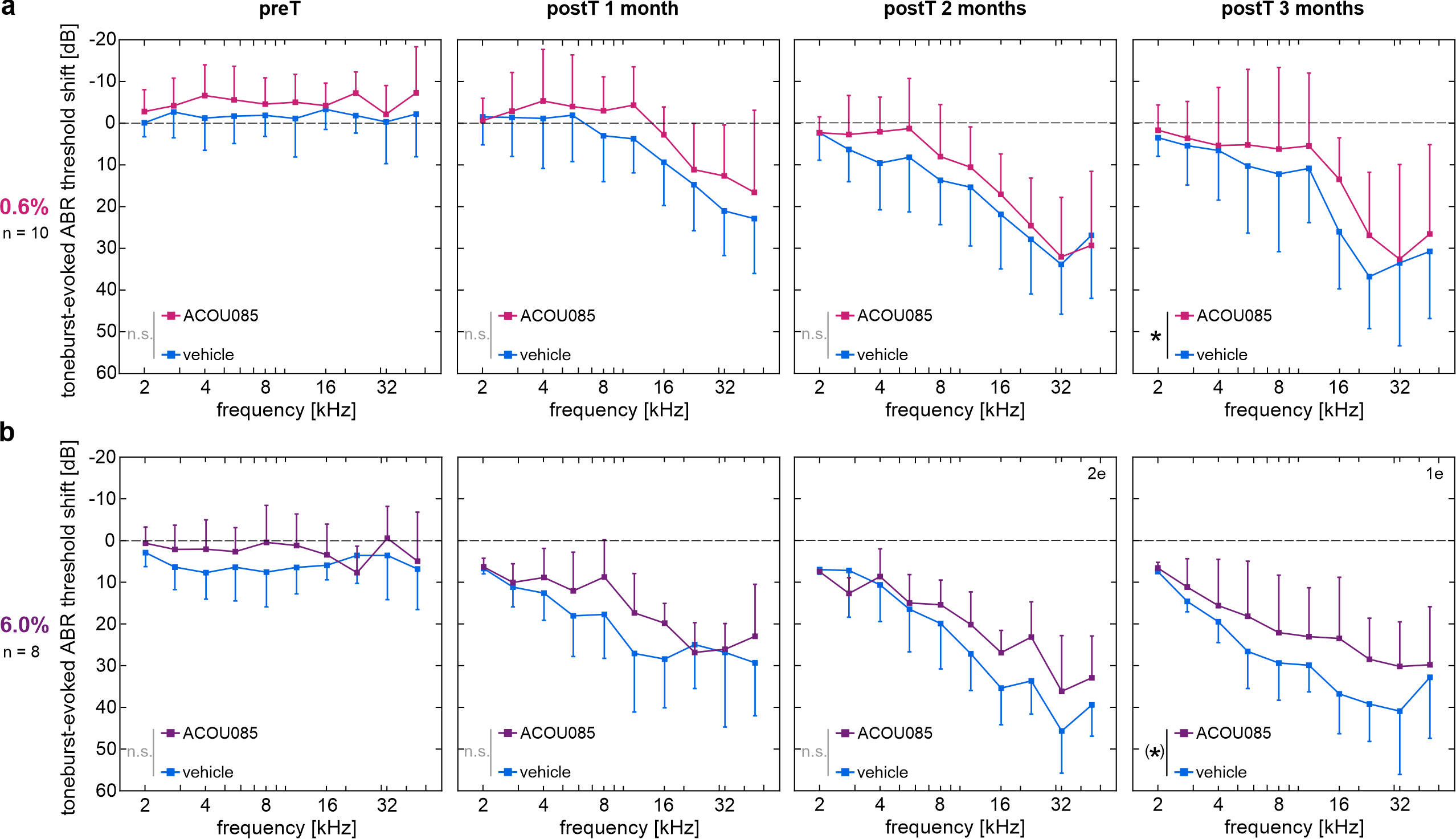

Supplement: Supplementary file 3 — Figure S2 [file 41419_2022_4915_MOESM3_ESM.png]
